# Efficacy of inverted internal limiting membrane flap for large idiopathic macular holes

**DOI:** 10.12669/pjms.35.2.689

**Published:** 2019

**Authors:** Haroon Tayyab, Asad Aslam Khan, Sana Jahangir

**Affiliations:** 1 *Dr. Haroon Tayyab, FCPS (Ophth), FCPS (VRO), Department of Ophthalmology, College of Ophthalmology & Allied Vision Sciences, King Edward Medical University, Lahore, Pakistan*; 2 *Prof. Asad Aslam Khan, MS, FCPS, PhD, Department of Ophthalmology, College of Ophthalmology & Allied Vision Sciences, King Edward Medical University, Lahore, Pakistan*; 3 *Dr. Sana Jahangir, FCPS, Department of Ophthalmology, Sharif Medical & Dental College, Lahore, Pakistan*

**Keywords:** Idiopathic macular hole, Internal limiting membrane, Pars plana vitrectomy

## Abstract

**Objective:**

To evaluate the effectiveness of inverted internal limiting membrane flap in large idiopathic macular holes.

**Methods:**

Twelve patients diagnosed with idiopathic macular holes larger than 400um underwent 23 gauge Pars Plana Vitrectomy (PPV) with inverted internal limiting flap and gas tamponade at Al-Ehsan Eye Welfare Eye Hospital, Lahore from February 2017 to February 2018. All cases were diagnosed on Spectral Domain Optical Coherence Tomography (SD-OCT) and were followed up for 6 months. At every follow-up, best corrected visual acuity and SD-OCT was done.

**Results:**

We achieved macular hole closure in 91.6% (11/12) patients with idiopathic macular holes larger than 400um. Five out of 12 patients underwent combined phacoemulsification and PPV. One patient has flat hole closure which was considered failure. One patient was excluded from the study due to per-operative flap loss. This patient was not included in final data analysis of 12 patients. There was statistically significant gain in best corrected visual acuity after successful macular hole closure. We did not report any untoward events during or in the post-operative period.

**Conclusion:**

Inverted internal limiting flap is an effective method for repairing large macular holes.

## INTRODUCTION

Idiopathic Macular Hole (IMH) is one of the most widely studied vitreoretinal interface pathology.^1^ Its reported incidence is 7.8 / 100,000 per annum with women being more commonly effected as compared to males.^1^,^2^ Before 1991, macular hole was considered an untreatable condition. It was Kelly and Wendel who for the first time reported Pars Plana Vitrectomy (PPV) and fluid gas exchange as a procedure of choice for IMH. They reported anatomical success in 58% of patient and functional success in 42% of patients in their pilot study.^3^

In further studies, they improved upon their results as their understanding of pathogenesis behind macular hole improved.^4^ Afterwards, various modifications were introduced in IMH surgery and the most notable was Internal Limiting Membrane (ILM) peeling. With ILM peeling, structural success of more than 90% was reported in various studies.^5^,^6^ Removal of ILM around the macular holes allows the retina to restore back to its inherent elastic state by removal of tangential centripetal traction around the hole edge. This technique has very good anatomic and functional results when the IMH minimum diameter is small.

When dealing with larger macular holes, the structural and functional success declines and also sometimes requiring reoperations.^7^ The most desirable macular hole closure is U-shaped closure with best visual results. In 20 to 40% of cases, surgeons achieve a W-shaped closure with exposed Retinal Pigment Epithelium (RPE). Although it is considered a structural success but visual gains are limited.^8^

In 2010, Michalewska introduced a novel technique in inverted ILM flap for closure of macular holes larger than 400um. He reported excellent structural and functional results later on. The rationale for his technique was to promote glial cell proliferation; thus plugging the macular hole and having some improvement in functional results too.^9^ As the technique gained popularity, more ambitious studies showed that inverted ILM peel technique was also successful in IMH larger than 1000um.^10^,^11^ These results showed that those macular holes that may have been left untreated due to their large size have a potential to benefit from this technique.

Other significant advances have been the introduction of different staining agents for staining ILM. The first one was indocyanine green dye followed by trypan blue and brilliant blue G dye. These vital dyes have made staining and peeling of ILM very easy and relatively safe in modern vitreoretinal surgery.^12^,^13^

Since there has been no local study to evaluate the results of inverted ILM peel in large macular holes, we attempted to assess the outcome in our local settings and share our experience regarding this technique.

## METHODS

This was a prospective interventional study conducted in vitreo-retina unit of Al- Ehsan Welfare Eye Hospital, Lahore, Pakistan (a tertiary care Ophthalmic facility) from February 2017 to February 2018. A total of 12 patients were enrolled. All patients were informed about the nature of disease and type of intervention being planned for their eyes. A written informed consent was obtained from all patients. Permission for this study was sought from Ethics Committee of the hospital. Patients with IMH larger than 400um were included in this study. Patients who had any other concurrent ocular pathology like retinal vascular diseases (diabetic retinopathy, retinal vein occlusion etc), age related macular degeneration, glaucoma, history of previous retinal surgery, history of trauma, uveitis, solar retinopathy and other vitreo-retinal interface problems were excluded from this study. Patients with high myopia (> 6 Diopter Sphere) or IMH with retinal detachment were also not included in this study. All phakic patients were simultaneously operated for cataract with foldable intraocular lens implant.

Best corrected visual acuity (BCVA) was recorded using Early Treatment Diabetic Retinopathy Study (ETDRS) type LogMAR chart. We evaluated IMH using spectral domain optical coherence tomography (Topcon 3D OCT-1 Maestro. Topcon Europe Medical BV Essebaan 11, 2908 LJ, Capelle aan den IJssel, The Netherlands). Macular holes with minimum diameter of > 400um were included in this study. Intraocular pressure was measured using standard Goldmann applanation tonometry (AT 900 – Applanation tonometer, Haag Streit AG, Germany). This examination was carried out before the surgery and 45 days after surgery to allow sufficient resolution of intraocular gas for convenient SD-OCT evaluation.

### Surgical Technique

All cases were performed using 23 gauge three port transconjunctival PPV by a single experienced vitreoretinal surgeon. Faros vitrectomy system (Oertli Instrumente AG, Switzerland) was used in all cases. After entry in vitreous cavity through 23 G trocar system implanted at 3.5mm from limbus, core vitrectomy was performed. We used 0.1 ml triamcinolone acetonide (40mg/ml) to stain posterior cortical vitreous and for induction of Posterior Vitreous Detachment (PVD). After complete vitrectomy, fluid air exchange was performed. Twin dye (TWIN 018 HD 0.18% trypan blue + 0.03% blulife, AL. CHI.MI.A. SRL - Viale Austria) was instilled on macula under air and left in place for 30 seconds for adequate staining. After washing out the dye and filling vitreous cavity with basic salt solution, ILM peel under high magnification was attempted. We used Oculus BIOM 2 with Oculus SDI Inverter 2 (OCULUS Surgical, Inc. Port St. Lucie, USA) as preferred viewing system in all cases. Grieshaber 23G disposable ILM peeling forceps were employed using pinch and peel technique for grasping and peeling ILM. ILM peel was started superiorly with a diameter of 2 discs around macular hole in such a way that only a thing rim remained attached to macular hole margins with a pedicle.

During this maneuver, perfusion was set at a low level. The ILM flap edges were trimmed with vitreous cutter using high cut rate (4000 cuts/min) and very low vacuum (50). Under low infusion pressures, ILM flap that remained attached to macular hole margins was massaged over the macular hole using silicon tip backflush needle in such a way that the ILM side facing the vitreous cavity was now facing RPE and covering entire area of exposed RPE. No extra manipulation was done here after. After positioning the flap in inverted manner over the macular hole, standard fluid air gas (GOT Multi C3F8 – pure octafluoropropane gas. AL.CHI.MI.A. SRL - - Viale Austria) exchange was performed with 14 % C3F8 filling vitreous cavity at the conclusion of surgery. Trocars were withdrawn and any visible gas leak was addressed with 7 0 Vicryl suture. Face down post-operative positioning was instructed for seven days. A follow up schedule of 1st week, 1st month, 2^nd^ month and 6^th^ month (total study time of one year from February 2017 to February 2018) was followed for every patient. Macular holes were considered closed if they were close at 6 months after surgery. Flat open closure type configuration was considered as surgical failure.

### Data analysis

For data analysis, we used SPSS software (version16.0; SPSS Inc., Chicago, Illinois, USA) for data analysis. Numerical data was represented as mean +/- standard deviation. Wilcoxon signed ranked test was used to assess statistical significance of pre and post-operative BCVA. P<0.05 was considered statistically significant.

## RESULTS

**Fig.1:**
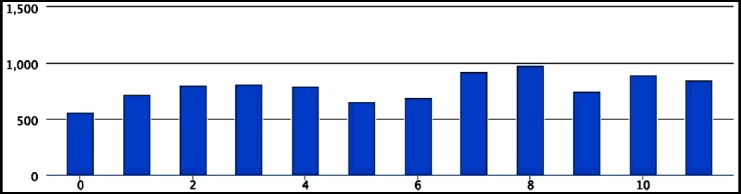
Shows minimum macular hole diameter in 12 study cases. X axis shows macular hole diameter in um.

A total of 12 eyes of 12 patients were included in this study. Eight (66.6%) were females and 4 (33.3%) were males. Mean age of patients in this study was 63.41± 5.93 years. Five out of 12 patients underwent simultaneous cataract extraction with foldable IOL implant. Using SD-OCT, the mean minimum width of IMH was 788 ± 120.73 um with range of 561 – 983 um. Individual values are shown in Fig.1. The mean duration of macular hole was 7.16 ±2.29 months. The mean BCVA before surgery was 1.236 ± 0.265 Log MAR units. Mean BCVA 2 months and 6 months after surgery was 0.989 ± 0.298 LogMAR units and 0.918±0.336 The difference between pre and post-operative BCVA at 2 months and 6 months was statistically significant (p-value < 0.05).

**Fig.2:**
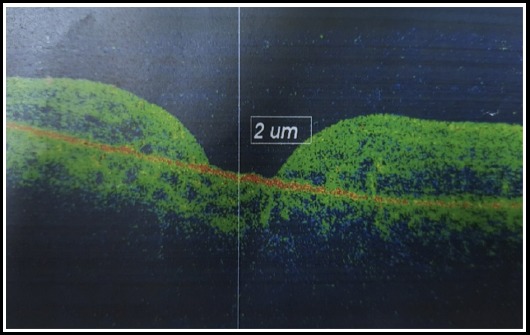
Type 2 macular hole closure.

**Fig.3:**
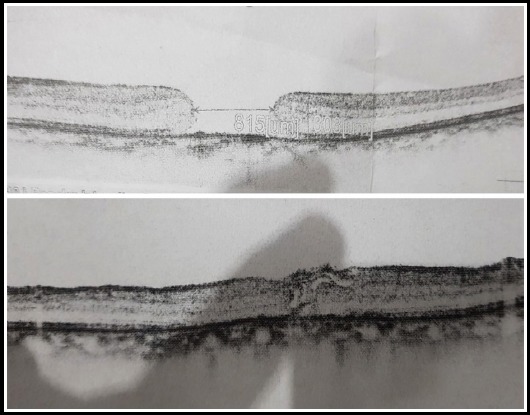
Shows pre-opertaive and post-operative OCTs of macular hole closure with inverted ILM flap.

**Fig.4:**
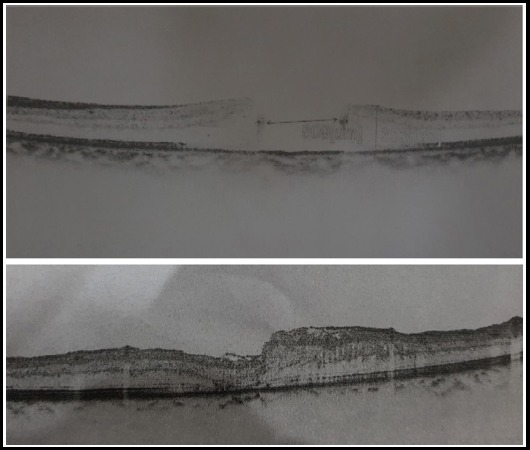
Shows pre-opertaive and post-operative OCTs of macular hole closure with inverted ILM flap.

We had structural success in 11 out of 12 (91.66%) cases. One IMH had Type-2 macular hole closure with flat macular hole edges but no retinal or glial tissue growth with exposed RPE (Fig.2). One case had loss of ILM flap during surgery and was not included in final analysis because only conventional ILM peel was accomplished in this case. We do not report any untoward events or side effects during or in the post-operative recovery period. Few cases with successful macular hole closure with inverted ILM flap are shown in Fig.3 and 4 with pre operative and post operative OCTs stacked on top and bottom of figures respectively. Follow up period for post op OCTs was two months.

## DISCUSSION

It has been known through literature and experience that larger macular holes have relatively lower rate of successful closure and visual improvement and higher rates of reopening.^14^-^16^ It was Michalewska in 2010 who reported a novel approach of inverted ILM flap for closing macular holes larger than 400um. He also reported significant visual improvement in his cases.^9^ Since then; there have been various reports on inverted ILM flap with or without some modifications. These modifications include pedicle ILM flap, autologous and pedicle ILM flap and double ILM flap.^17^-^19^ Surgeons have also successfully attempted inverted ILM flaps in cases of myopic macular holes and macular hole associated rhegmatogenous retinal detachment.^16^,^20^ In all these studies, researchers have taken a hole larger than 400um as large macular hole. Our inclusion criteria have also been the same. All patients included in this study had macular hole larger than 400um; although the IMH with minimum most diameter in this study was 561um. In a recent major review by Yamashita and Japan Clinical Retina Research Team (J-CREST), they reported 100% success rate for macular hole closure with inverted ILM flap as compared to conventional ILM peel. They reported 86% (38/43) closure rate for holes >550um with conventional ILM peel as compared to 100% (41/41) closure rate with inverted ILM flap. Their BCVA results are also comparable to our study.^16^ In another subgroup (macular holes >700um), the same team reported 69% (9/13) closure rate as compared to 100% (12/12) in conventional vs. inverted ILM flap respectively.

In another large series published by Rizzo recently, he showed 96% success in inverted ILM flap group versus 79% success in conventional group for IMH larger than 400um. They also reported similar success rate in BCVA as well.^21^ They also reported statistically significant results in favour of inverted ILM flap technique when considering eye with axial length >26mm (88% versus 39%).

Similar results were reported in smaller series when Khodani reported 100% (5/5) results with inverted ILM flap in macular holes with mean base diameter of 1420+/-84.8um.^11^ Mahalingham also reported 100% (5/5) results with inverted ILM flap technique with mean hole diameter of 811.4um.^10^

It was postulated by Michalewska that ILM flap provides mesh for proliferation of glial cells that eventually close the macular hole. This has been observed in many studies conducted in inverted macular flaps. It was further proposed after wards that the inverted flap not only provides a scaffold for glial cells to proliferate but also some stimulates the movement of photoreceptors towards the centre of fovea; thus providing neurosensory retina in the area of exposed RPE.^9^ In few of the cases it was shown in our study that inverted ILM flap actually stimulated the centripetal movement of photoreceptors to close the macular hole. (Fig.3 and 4).

In our study, we had structural success in 11/12 (91.66%) cases with mean smallest diameter of macular hole being 788um. We also report statically significant BCVA improvement. The strength of our study was its prospective design and a constant variant of ILM flap technique being performed by single surgeon with adequate follow-up period. The deficiencies include its small sample size with lack of control arm.

## CONCLUSION

After extensive literature review and observation of our own results, we conclude that vitreoretinal surgeon should consider inverted ILM flap for large macular holes (>400um). Although, this technique is a brief learning curve but it can yield excellent anatomic outcome for macular holes previously considered as high risk for failure after undergoing conventional ILM peel. We recommend studies with multiple centres and more patients to evaluate the efficacy of this technique in our local setups. We also need to conduct study with control group undergoing conventional ILM peel for large macular holes to have more meaningful statistical results.

### Author`s Contribution

**HT** primary surgeon & conceived the design.

**AAK** did data collection and manuscript writing.

**SJ** did review of literature and proof reading.
